# Basophil Activation Test Reduces Oral Food Challenges to Nuts and Sesame

**DOI:** 10.1016/j.jaip.2020.12.039

**Published:** 2021-05

**Authors:** Alexandra F. Santos, Marcel Bergmann, Helen A. Brough, Natália Couto-Francisco, Matthew Kwok, Valentina Panetta, Diab Haddad, Gideon Lack, Philippe Eigenmann, Jean-Christoph Caubet

**Affiliations:** a Department of Women and Children's Health (Pediatric Allergy), School of Life Course Sciences, Faculty of Life Sciences and Medicine, King's College London, London, United Kingdom; b Peter Gorer Department of Immunobiology, School of Immunology and Microbial Sciences, King's College London, London, United Kingdom; c Children's Allergy Service, Evelina London, Guy's and St Thomas' Hospital, London, United Kingdom; d Asthma UK Centre in Allergic Mechanisms of Asthma, London, United Kingdom; e Pediatric Allergy Unit, Department of Pediatrics, University Hospitals of Geneva, Geneva, Switzerland; f L'altrastatistica srl, Consultancy & Training, Biostatistics Office, Rome, Italy; g Department of Pediatrics, St Peters' Hospital, London, United Kingdom

**Keywords:** Food allergy, Basophil activation test, Tree nuts, Sesame seed, Peanut, Skin prick test, Specific IgE, Diagnosis, Severity, Threshold dose, BAT, Basophil activation test, NPV, Negative predictive value, OFC, Oral food challenge, PPV, Positive predictive value, ROC, Receiver operating characteristic, sIgE, Specific IgE, SPT, Skin prick testing

## Abstract

**Background:**

Nut allergic patients are often IgE sensitized to other nuts/seeds and need multiple oral food challenges (OFCs) before the safe nuts can be introduced in the diet. However, OFCs are time-consuming and risky procedures.

**Objective:**

To assess the utility of the basophil activation test (BAT) to predict the allergic status and reduce the need for an OFC in children with 1 or more nut or seed allergies.

**Methods:**

Participants in the Pronuts study recruited at the Geneva and the London centers were tested on the BAT to hazelnut, cashew nut, sesame, almond, and peanut, Ara h 1, Ara h 2, Ara h 6, using FlowCAST, a commercially available BAT kit, and flow cytometry.

**Results:**

The BAT to hazelnut, cashew nut, sesame, almond, and peanut discriminated between allergic and nonallergic children, to the respective nut or seed. The optimal allergen concentration and their optimal, positive, and negative cutoffs were identified for the BAT and the other tests, for each nut and seed. Using the BAT as a second step in the diagnostic process, after equivocal skin prick test and IgE to extracts and components, reduced the number of total OFCs by 5% to 15% and positive OFCs by 33% to 75% (except for hazelnut) with 0% false-negatives and a diagnostic accuracy of 96% to 100%.

**Conclusion:**

The BAT proved to be a useful diagnostic tool, used in a stepwise approach, to predict the allergic status and reduce the number of OFCs in the Pronuts study participants with at least 1 nut allergy willing to consume selected nuts.

***What is already known about this topic?*** The introduction of nuts and seeds in the diet of children with 1 or more nut allergies is safe and feasible; however, because of polysensitization, this often requires multiple oral food challenges (OFCs).

***What does this article add to our knowledge?*** The basophil activation test (BAT), when used after skin prick and specific IgE testing, can reduce the number of OFCs, particularly positive OFCs, maintaining very high diagnostic accuracy.

***How does this study impact current management guidelines?*** In children with 1 or more nut allergies, needing an OFC to clarify the allergic status to other nuts, a positive BAT confirms allergy, whereas a negative BAT requires an OFC before recommending nut consumption or avoidance.

IgE sensitization to tree nuts and seeds is common in children with peanut and other nut and seed allergies and does not necessarily translate into clinical reactivity.^1^^,^^2^ Tree nut and seed allergies can lead to not only dietary but also social restrictions and significant anxiety associated with the fear of developing potentially severe allergic reactions unexpectedly.^3^^,^^4^ This has wider implications in the lives of children and their families and can significantly impact on their quality of life.^3^, ^4^, ^5^ A significant proportion of children allergic to 1 or more nuts or seeds are able to tolerate other nut(s).^2^ In motivated families, interested and able to consume selected nuts while avoiding others, the allergic status to individual nuts and seeds can be verified and selective consumption of the nuts to which there is proven tolerance can be encouraged.^6^, ^7^, ^8^ This should be accompanied by comprehensive information about potential risks, namely cross-contamination and misidentification of nuts, and the need to continue regular consumption of the safe nuts at home.^9^ The Pronuts study recently demonstrated that the introduction of nuts and seeds in the diet of children with 1 or more nut allergies is safe and feasible.^2^

Fear of coallergy in children allergic to 1 or more nuts frequently leads to blanket advice to avoid all nuts. Concerns regarding potential allergy to nuts also arise when managing children with other food allergies, with a family history of nut allergies and/or with underlying atopic conditions. The demonstration of sensitization to nuts on skin prick testing (SPT) or specific IgE (sIgE) testing can heighten such concerns. Although nonsensitized children without a history of reaction are often recommended to introduce the nuts in the diet at home, sensitized children might have to undergo an oral food challenge (OFC) and often multiple OFCs to allow safe consumption of nuts and seeds that children are not allergic to.^2^ Given the risk and resources involved in the performance of an OFC, it would be beneficial to have a diagnostic approach that could reduce the number of children requiring an OFC and allow the proactive introduction of safe nuts in the diet.

The basophil activation test (BAT) is a flow cytometry–based test that assesses the expression of activation markers, namely CD63, on the surface of blood basophils after stimulation with allergen or controls.^10^ We previously demonstrated that the BAT to peanut had 97% diagnostic accuracy and could reduce the number of children requiring an OFC by approximately 67%.^11^ We have further validated the diagnostic utility of the BAT in a large prospectively independent study of well-characterized patients.^12^ Considering the high specificity of the BAT and the practicalities involved in its performance (eg, BAT requires fresh blood and flow cytometry), we have proposed that the BAT could be used as a second step in the diagnosis of food allergy, in patients for whom the combination of the clinical history with SPT or IgE testing could not lead the clinician to a definite diagnosis.^13^^,^^14^

In this substudy of the Pronuts study, we aimed to assess the utility of the BAT, using a commercially available kit, to diagnose nut and seed allergies in patients with at least 1 nut or seed allergy and the impact of the BAT on the number of OFCs required to reach an accurate diagnosis and enable the clinician to provide appropriate advice on avoidance or consumption of nuts or seeds. We hypothesized that the BAT had high diagnostic accuracy and allowed reduction in the number of OFCs required, thus leading to a more accurate and safe approach to diagnosing tree nut and seed allergies.

## Methods

### The Pronuts study

The Pronuts study (NCT01744990 in Clinicaltrials.gov) was a prospective multicenter study, with recruitment undertaken between 2012 and 2015, which aimed to assess safety and feasibility of introducing nuts in the diet of children with at least a single nut allergy. The method is described extensively elsewhere.^2^ Briefly, children aged between 6 months and 16 years seen in specialized Pediatric Allergy centers in London, Geneva, and Valencia were invited to participate. At the core of the recruitment was the confirmation of the diagnosis of allergy to at least 1 nut, including peanut, sesame, and tree nuts. Diagnosis of allergy was confirmed by positive OFCs or a convincing history of IgE-mediated allergic reaction to the culprit nut in the previous 12 months and SPT and sIgE greater than or equal to the 95% positive predicting value for the respective nut or seed allergy (eg, 8 mm on SPT and 15 kU/L on sIgE to peanut^11^^,^^15^). Exclusion criteria were uncontrolled asthma, chronic urticaria, chronic systemic disease, daily antihistamine or oral allergy syndrome only to the index nut, history of life-threatening anaphylaxis as defined by documented desaturation <89%, >20% drop systolic in blood pressure, or admission to a pediatric intensive care unit (other cases of anaphylaxis were admissible). Ethical approval was obtained at each site, namely UK (14/LO/0066), Geneva (CER 12-020PS), and Valencia (2012/0108), and written informed consent was obtained from all participants.

### Study procedures

Children screened for entry into the study underwent clinical assessment, SPT, blood collection for sIgE testing, and BAT and OFCs. For each nut/seed, 3 groups of patients were defined based on the allergic status (allergic vs nonallergic) and on the presence of allergen-specific IgE: sensitized allergic, sensitized nonallergic, and nonsensitized nonallergic. The clinical information, SPT, and OFC results were not available to the performers of sIgE or BAT. Clinical information and SPT results were available to the team performing OFCs. As this substudy focuses on the utility of the BAT to peanut, sesame, cashew, hazelnut, and almond and the BAT was performed only at the London and Geneva sites, the analyses presented here are limited to data acquired at these 2 study sites and for the aforementioned nuts and seeds.

### Skin prick testing and specific IgE measurements

SPT was performed using Stallerpoint plastic lancets (Stallergenes, Antony, France) and commercial allergen extracts for peanut, hazelnut, cashew, and almond (Stallergenes, Antony, France) and tahini paste (Meridian Foods, Hampshire, UK) for sesame. Maximum wheal diameter was recorded after 15 minutes.

Serum sIgE levels to allergen extracts (cashew nut, sesame, hazelnut, almond, and peanut) and to individual allergens (Ara h 1/2/3/8/9, Cor a 1/8/9/14, and Ana o 3) were measured using ImmunoCAP (ThermoFisher Scientific, Uppsala, Sweden).

### Basophil activation test

The BAT was performed to hazelnut, cashew nut, sesame, almond, and peanut extracts and peanut components Ara h 1, Ara h 2, Ara h 6, using stimulants (CAST allergens; Basel, Switzerland) and reagents provided in the Flow CAST kit (BÜHLMANN, Basel, Switzerland) and following the manufacturer's instructions. A schematic figure of the BAT procedure has been included in a previous publication.^13^ Briefly, blood was collected in an EDTA-containing Vacutainer tube and mixed gently. Stimulation and lysing buffers were prewarmed to room temperature. Allergens were diluted following the allergen-dilution scheme shown in Table E1 (available in this article's Online Repository at www.jaci-inpractice.org). Equal volume (50 μL) of stimulant and whole blood and 100 μL of stimulation buffer were added to 5 mL tubes and mixed gently. Staining reagent (20 μL) containing anti-CCR3-PE and anti-CD63-FITC was added subsequently. All tubes were mixed, covered, and incubated at 37°C for 25 minutes in an incubator, after which 2 mL of lysing reagent was added and each tube vortexed gently and incubated for 10 minutes at room temperature in the dark. After centrifugation at 500 × *g* for 5 minutes, supernatants were decanted gently and pellets resuspended and kept at 4°C until analyses. Flow cytometry was performed at each site in a FACS CantoII with FACSDiva software (BD Biosciences, San Jose, Calif), and data were analyzed using FlowJo software (version 7.6.5; TreeStar, Ashland, Ore) by an investigator who was blinded to the clinical features of the participants. Basophils were gated as SSClow/CCR3+ and activation was expressed as %CD63+ basophils, corrected for the spontaneous basophil activation (ie, subtracted the %CD63+ basophils in the unstimulated condition). All the flow cytometry data were analyzed by the same researcher at the London center who was blind to all the clinical features. Reagents for the BAT were provided by BÜHLMANN under agreements with King's College London and Geneva University Hospitals.

### Oral food challenges

OFCs were unblinded and performed following the PRACTALL guidelines reaching a cumulative dose of 4.43 g of nut protein for patients of 36 months of age or older and 3.43 g for younger children. Allergic reactions were treated according to the local hospital guidelines. Children with positive OFCs were recommended to avoid the nut strictly in the diet and provided with an emergency treatment plan, whereas children with negative OFCs were recommended to consume the nut regularly in the diet.

### Statistical analyses

**Figure 1:**
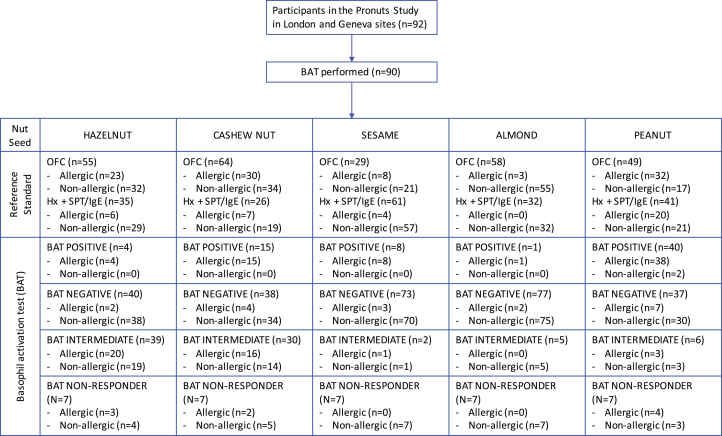
Consort diagram. BAT allergen stimulants used in this diagram were 4.545 ng/mL hazelnut extract, 22.73 ng/mL cashew nut extract, 113.64 ng/mL sesame extract, 113.64 ng/mL for almond, and 4.55 ng/mL Ara h 2, all CAST allergens. *BAT*, Basophil activation test; *Hx*, clinical history; *OFC*, oral food challenge; *SPT*, skin prick test.

Qualitative variables were reported as number and percentage and compared using the χ^2^ test. The χ^2^ test was also used to compare all categorical variables. Quantitative variables were reported as median and interquartile range and compared using the Mann-Whitney and Kruskal-Wallis tests for 2 or more than 2 groups, respectively. Receiver operating characteristic (ROC) analyses were used to assess the discriminative ability of tests between allergic and nonallergic subjects. Optimal concentration of allergen for the BAT was determined based on the largest area under the ROC curve. Comparison of ROC curves was made by the DeLong test included in the SAS ROCCONTRAST Statement.^16^ Optimal, negative, and positive cutoffs were determined based on the Youden index, 95% negative predictive value (NPV), and 95% positive predictive value (PPV). Cutoffs generated based on this dataset were used to determine the equivocal cases when assessing the diagnostic workup in 2 steps. Seven (7.8%) subjects had nonresponder basophils and were excluded from the ROC curve analyses as were subjects without results for the other tests as only subjects with complete datasets could be included. Demographic and clinical characteristics of these 7 patients did not differ from the rest of the population (Table E2, available in this article's Online Repository at www.jaci-inpractice.org). In the real-life assessment of the BAT used as a second step in the diagnostic process, subjects with nonresponder basophils were included. For all tests, including the BAT, results at or above the 95% PPV cutoff were considered positive; results below the 95% NPV were considered negative and the results between cutoffs were considered equivocal. The impact in the number of OFCs was calculated as if all patients had undergone OFCs with the outcome of OFCs based on the allergic status (Figure 1) and a subanalysis was performed considering only the participants that underwent OFC as part of the Pronuts study protocol (Table IV). SAS 9.4 was used for all analyses; a *P* value <.05 was considered statistically significant.

## Results

### Study population

Ninety-two children were assessed for possible allergy to cashew, hazelnut, almond, peanut, and sesame seed at the London and Geneva centers and 90 (98%) were tested on the BAT to all 5 foods. The consort diagram in Figure 1 shows the definition and outcome of reference standard and the outcome of the BAT for each nut or seed. Demographic, clinical, and immunologic characteristics of the studied population are reported in Table I. The prevalence of cosensitizations and coallergies to different nuts was previously published for the whole Pronuts study cohort.^2^ Overall, the most common allergy in the cohort studied here was peanut allergy followed by cashew nut, hazelnut, sesame seed, and almond allergies. Cashew nut allergy was more common in Geneva, but the prevalence of atopic comorbidities, namely eczema, asthma, and allergic rhinitis, was similar across centers. Children seen in London were slightly younger and showed a higher proportion of activated basophils in response to peanut, Ara h 2, and the IgE-mediated positive control anti-FcεRI (but not the non–IgE-mediated positive control fMLP) compared with children seen in Geneva.

**Table I:** Demographic and clinical characteristics of participants in this substudy of the Pronuts study

Clinical characteristics	Study population (n = 90)	GB (n = 49)	GE (n = 41)	*P* value
Age (y)	5.1 (3-9)	4.4 (2-8)	5.8 (4-10)	**.031**
Gender (male), % (n)	54.4 (49)	55.1 (27)	53.7 (22)	.891
Atopic eczema, % (n)	61.1 (55)	63.3 (31)	58.5 (24)	.647
Allergic rhinitis, % (n)	46.7 (42)	42.9 (21)	51.2 (21)	.428
Asthma, % (n)	32.2 (29)	24.5 (12)	41.5 (17)	.086
Other food allergy, % (n)	41.1 (37)	44.9 (22)	36.6 (15)	.425
Nut and seed allergies, % (n)				
Hazelnut allergy	32.2 (29)	30.6 (15)	34.1 (14)	.721
Cashew nut allergy	41.1 (37)	28.6 (14)	56.1 (23)	**.008**
Sesame seed allergy	13.3 (12)	14.3 (7)	12.2 (5)	.771
Almond allergy	3.3 (3)	2.0 (1)	4.9 (2)	.455
Peanut allergy	57.8 (52)	63.3 (31)	51.2 (21)	.249

*GB*, Great Britain site; *GE*, Geneva site.

Median (interquartile range) for quantitative variables. *P* < .05 were considered significant and marked in bold.

### Basophil activation test discriminated peanut, tree nut, and seed allergic from nonallergic children

**Figure 2:**
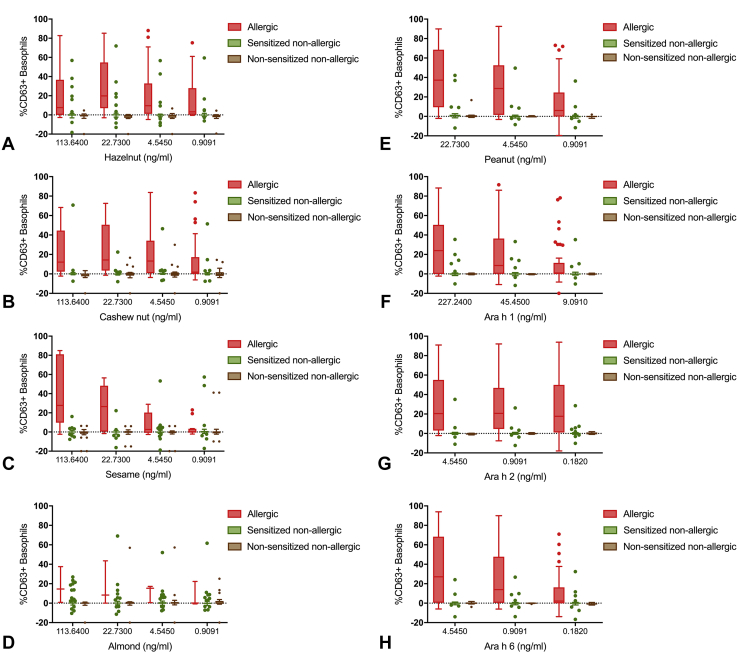
Basophil activation to tree nuts, sesame, peanut or their allergen components in allergic (in red), sensitized nonallergic (in green), and nonsensitized nonallergic children (in brown). n = 83 (7 participants with nonresponder basophils were excluded). (**A**) Hazelnut. (**B**) Cashew. (**C**) Sesame. (**D**) Almond. (**E**) Peanut. (**F**) Ara h 1. (**G**) Ara h 2. (**H**) Ara h 6.

**Figure E1:**
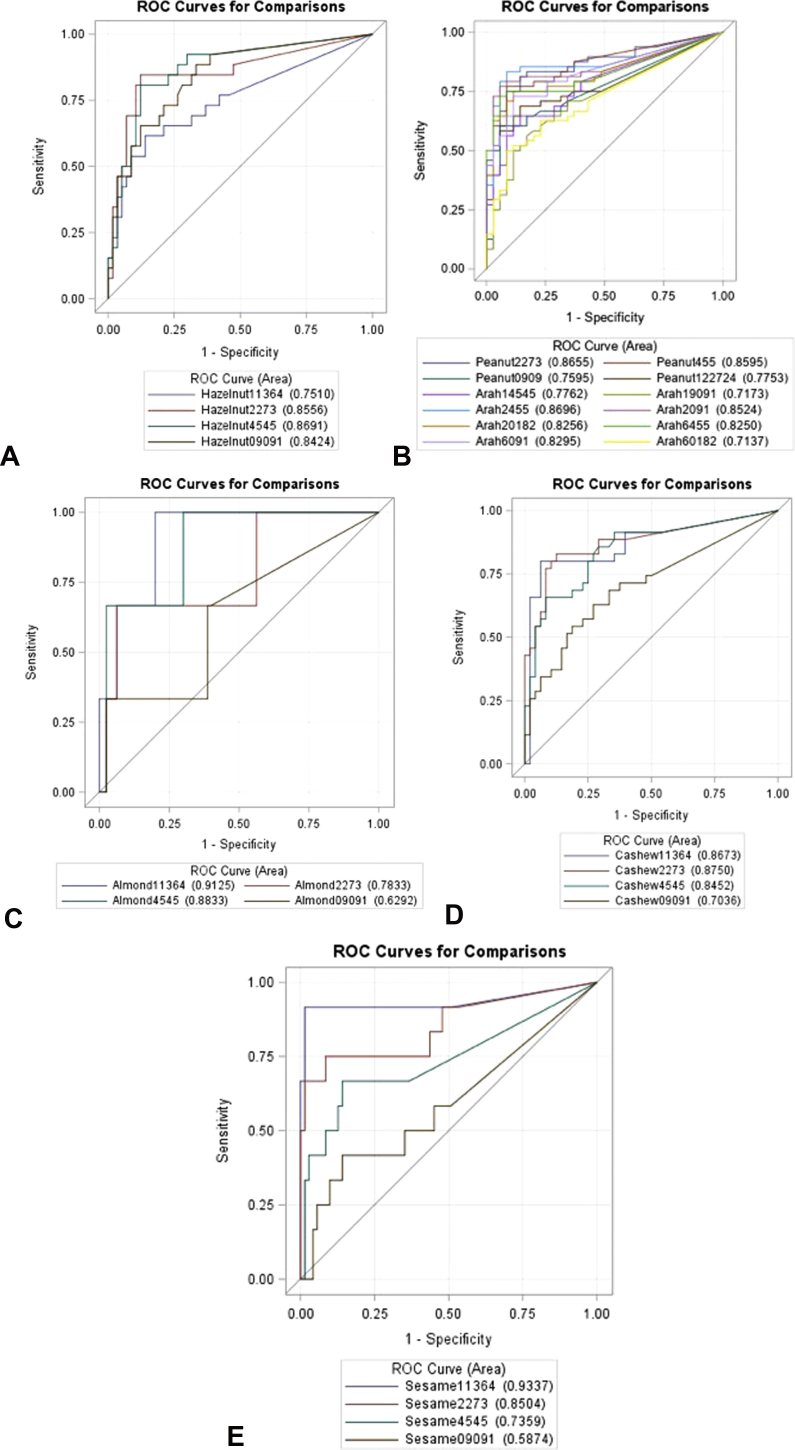
ROC curve analyses of the basophil activation test at different allergen concentrations to diagnose peanut, tree nut, and sesame seed allergies. (**A**) Hazelnut. (**B**) Peanut. (**C**) Almond. (**D**) Cashew. (**E**) Sesame. *ROC*, Receiver operating characteristic.

The BAT to hazelnut, cashew nut, sesame, almond, peanut, Ara h 1, Ara h 2, and Ara h 6 showed a higher proportion of activated basophils in allergic compared with nonallergic subjects (Figure 2 and Table II) (*P* < .001 in the vast majority of allergen concentrations). Ara h 2 on the BAT performed better than Ara h 6, Ara h 1, or peanut extract. For each nut, an optimal allergen concentration was identified based on the largest area under the ROC curve built for the discrimination between allergy and tolerance (Figure E1, available in this article's Online Repository at www.jaci-inpractice.org). Optimal concentrations of allergen tested were 22.73 ng/mL for peanut, 45.45 ng/mL for Ara h 1, 24.55 ng/mL for Ara h 2, 0.91 ng/mL for Ara h 6, 4.545 ng/mL for hazelnut, 22.73 ng/mL for cashew, 113.64 ng/mL for almond, and 113.64 ng/mL for sesame.

**Table II:** Immunologic characteristics of allergic and nonallergic subjects (n = 83)

	Nonallergic	Allergic	*P* value	AUC ROC (95% CI)		
Hazelnut allergy	N = 57	N = 26				
SPT weal diameter (mm)	0.0 (0-4)	10.0 (6-14)	**<.001**	0.8721	0.7984	0.9458
Specific IgE (kU/L)						
Hazelnut	0.64 (0.1-3.8)	6.45 (2.5-18.5)	**<.001**	0.7763	0.6764	0.8762
Cor a 1	0.01 (0.0-2.1)	0.57 (0.0-10.7)	**.026**	0.6495	0.5205	0.7785
Cor a 8	0.01 (0.0-0.0)	0.02 (0.0-0.1)	.058	0.6082	0.4784	0.7380
Cor a 9	0.13 (0.0-0.9)	4.20 (0.3-8.8)	**<.001**	0.7390	0.6173	0.8608
Cor a 14	0.02 (0.0-0.1)	3.27 (0.3-16.0)	**<.001**	0.8659	0.7717	0.9600
Basophil activation test (%CD63+ basophils)						
Hazelnut 113.64 ng/mL	0.0 (0-1)	7.7 (0-36)	**<.001**	0.7510	0.6301	0.8719
Hazelnut 22.73 ng/mL	0.0 (0-0)	19.8 (8-52)	**<.001**	0.8556	0.7558	0.9554
Hazelnut 4.545 ng/mL	0.0 (0-0)	9.6 (1-31)	**<.001**	0.8691	0.7831	0.9551
Hazelnut 0.9091 ng/mL	0.0 (0-0)	3.2 (0-26)	**<.001**	0.8424	0.7519	0.9330
Cashew nut allergy	N = 48	N = 35				
SPT weal diameter (mm)	0.0 (0-2)	12.0 (9-15)	**<.001**	0.9762	0.9422	1.0000
Specific IgE to cashew (kU/L)	0.19 (0.0-0.7)	4.15 (1.1-10.8)	**<.001**	0.8867	0.8148	0.9587
Specific IgE to Ana o 3 (kU/L)	0.01 (0.0-0.1)	3.89 (0.9-10.7)	**<.001**	0.9737	0.9417	1.0000
Basophil activation test (%CD63+ basophils)						
Cashew 113.64 ng/mL	0.0 (0-1)	12. (2-45)	**<.001**	0.8673	0.7798	0.9548
Cashew 22.73 ng/mL	0.0 (0-1)	14.4 (3-51)	**<.001**	0.8750	0.7939	0.9561
Cashew 4.545 ng/mL	0.0 (0-1)	13.3 (1-34)	**<.001**	0.8452	0.7577	0.9328
Cashew 0.9091 ng/mL	0.0 (0-1)	1.9 (0-17)	**.001**	0.7036	0.5892	0.8180
Almond allergy	N = 79	N = 3				
SPT weal diameter (mm)	0.0 (0-2)	8.0 (3-12)	**.005**	0.8945	0.7028	1.0000
Specific IgE to almond (kU/L)	0.20 (0.1-1.3)	1.64 (1.5-2.8)	.065	0.8143	0.7250	0.9037
Basophil activation test (%CD63+ basophils)						
Almond 113.64 ng/mL	0.1 (0-1)	14.5 (1-38)	**.013**	0.9125	0.7895	1.0000
Almond 22.73 ng/mL	0.2 (0-1)	8.6 (0-44)	.085	0.7833	0.4402	1.0000
Almond 4.545 ng/mL	0.1 (0-1)	15.3 (1-17)	**.018**	0.8833	0.6981	1.0000
Almond 0.9091 ng/mL	0.0 (0-1)	0.1 (0-22)	.396	0.6292	0.2427	1.0000
Sesame seed allergy	N = 71	N = 12				
SPT weal diameter (mm)	0.0 (0-1)	12.5 (8-21)	**<.001**	0.9137	0.7969	1.0000
Specific IgE to sesame (kU/L)	0.30 (0.1-2.3)	3.10 (1.6-29.1)	**<.001**	0.8173	0.7140	0.9205
Basophil activation test (%CD63+ basophils)						
Sesame 113.64 ng/mL	0.0 (0-0)	27.7 (11-79)	**<.001**	0.9337	0.8109	1.0000
Sesame 22.73 ng/mL	0.1 (0-1)	26.6 (1-48)	**<.001**	0.8504	0.7004	1.0000
Sesame 4.545 ng/mL	0.0 (0-1)	2.7 (0-16)	**.003**	0.7359	0.5552	0.9166
Sesame 0.9091 ng/mL	0.0 (0-1)	0.3 (0-3)	.306	0.5874	0.3961	0.7788
Peanut allergy	N = 35	N = 48				
SPT weal diameter (mm)	0.0 (0-3)	10.5 (9-15)	**<.001**	0.9314	0.8734	0.9893
Specific IgE (kU/L)						
Peanut	0.35 (0.1-2.1)	14.60 (3.4-50.9)	**<.001**	0.8984	0.8328	0.9639
Ara h 1	0.01 (0.0-0.1)	0.72 (0.0-11.9)	**<.001**	0.7696	0.6686	0.8706
Ara h 2	0.01 (0.0-0.1)	10.80 (1.6-33.6)	**<.001**	0.9536	0.9033	1.0000
Ara h 3	0.03 (0.0-0.1)	0.13 (0.0-1.6)	**.028**	0.6222	0.4993	0.7451
Ara h 8	0.03 (0.0-1.0)	0.01 (0.0-1.3)	.406	0.5585	0.4352	0.6817
Ara h 9	0.01 (0.0-0.1)	0.01 (0.0-0.0)	.155	0.3768	0.2588	0.4948
Basophil activation test (%CD63+ basophils)						
Peanut 22.73 ng/mL	0.3 (0-1)	37.3 (10-68)	**<.001**	0.8655	0.7862	0.9447
Peanut 4.55 ng/mL	0.0 (0-0)	28.7 (2-53)	**<.001**	0.8595	0.7810	0.9381
Peanut 0.909 ng/mL	0.0 (0-0)	6.1 (0-24)	**<.001**	0.7595	0.6621	0.8570
Ara h 1 22.724 ng/mL	0.0 (0-1)	24.0 (0-50)	**<.001**	0.7753	0.6780	0.8726
Ara h 1 4.545 ng/mL	0.0 (0-1)	8.6 (0-36)	**<.001**	0.7762	0.6807	0.8717
Ara h 1 0.9091 ng/mL	0.0 (0-0)	1.0 (0-11)	**<.001**	0.7173	0.6119	0.8226
Ara h 2 4.55 ng/mL	0.0 (0-0)	20.5 (3-53)	**<.001**	0.8696	0.7891	0.9502
Ara h 2 0.91 ng/mL	0.0 (0-0)	20.7 (5-47)	**<.001**	0.8524	0.7686	0.9362
Ara h 2 0.182 ng/mL	0.0 (0-1)	17.7 (2-50)	**<.001**	0.8256	0.7376	0.9136
Ara h 6 4.55 ng/mL	0.0 (0-0)	27.1 (1-67)	**<.001**	0.8250	0.7373	0.9127
Ara h 6 0.91 ng/mL	0.0 (0-0)	14.0 (0-48)	**<.001**	0.8295	0.7459	0.9130
Ara h 6 0.182 ng/mL	0.0 (0-0)	2.1 (0-61)	**<.001**	0.7137	0.6084	0.8190

*AUC*, Area under the curve; *CI*, confidence interval; *ROC*, receiver operating characteristic; *SPT*, skin prick testing.

Median and interquartile range are represented. Subjects with nonresponder basophils were excluded. *P* < .05 were considered significant and marked in bold.

**Figure 3:**
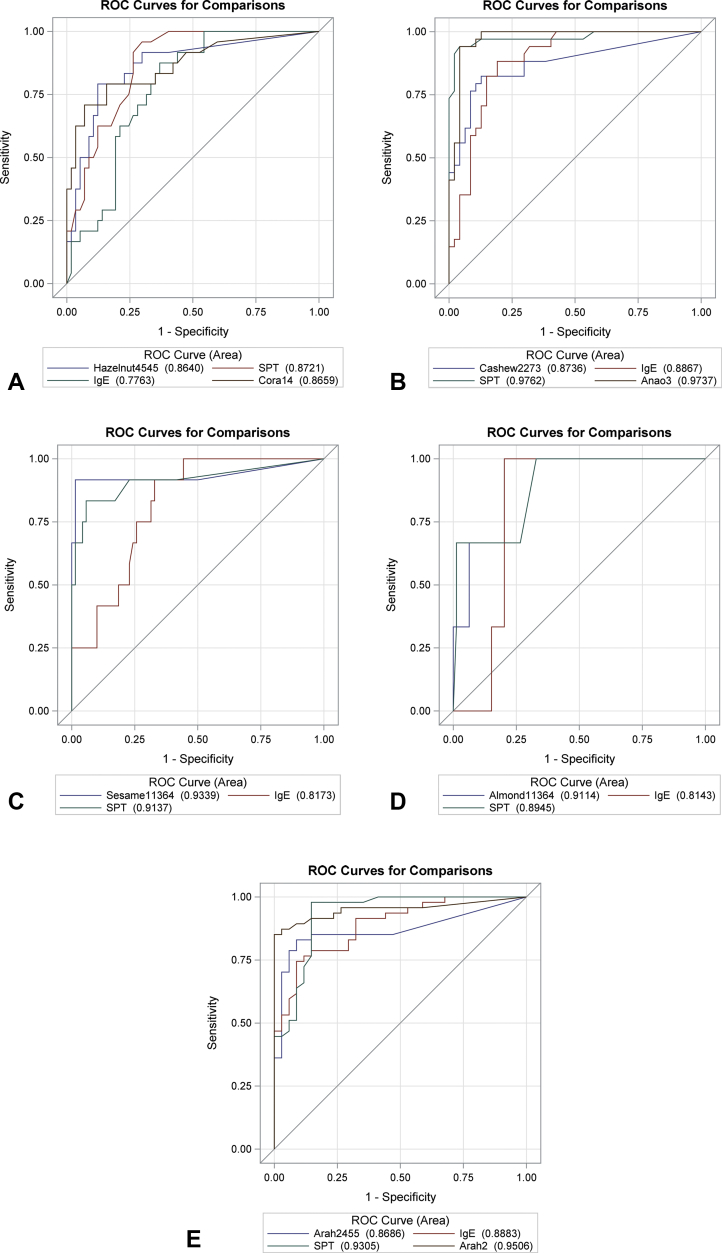
Receiver operating characteristic (ROC) curve for different tests for the various nut allergies. (**A**) Hazelnut allergy (*P* = .230 for comparison of areas under the ROC curves). (**B**) Cashew nut allergy (*P* = .007 for comparison of areas under the ROC curves). (**C**) Sesame seed allergy (*P* = .215 for comparison of areas under the ROC curves). (**D**) Almond allergy (*P* = .232 for comparison of areas under the ROC curves). (**E**) Peanut allergy (*P* = .094 for comparison of areas under the ROC curves). *SPT*, Skin prick testing.

Based on ROC curve analyses, cutoffs were generated for the BAT to each nut or seed, including the optimal cutoff (ie, best balance between sensitivity and specificity determined by the Youden index), negative cutoff (ie, closest to the 95% NPV), and positive cutoff (ie, closest to the 95% PPV). The sensitivity, specificity, PPV, and NPV for each cutoff are indicated in Table III. Although not statistically significant except for cashew, the area under the ROC curve for the BAT was larger than the ones for the other available tests in the diagnosis of sesame and almond, similar for hazelnut and lower for peanut and cashew nut allergies (Figure 3).

**Table III:** Cutoffs for the basophil activation test to different nuts and their diagnostic performance (n = 83, nonresponders were excluded)

Allergen	Cutoff	Sensitivity (95% CI)	Specificity (95% CI)	Positive predictive value (95% CI)	Negative predictive value (95% CI)
BAT to hazelnut	60.98	15.38 (4.4-34.9)	100.00 (93.7-100)	100.00 (39.8-100)	72.15 (60.9-81.7)
	0.924	80.77 (60.7-93.5)	87.72 (76.3-94.9)	75 (55.1-89.3)	90.91 (80.0-97.0)
	0.13	92.31 (74.9-99.1)	66.67 (52.9-78.6)	55.81 (39.9-70.9)	95.00 (83.1-99.4)
BAT to cashew	25.21	42.86 (26.3-60.7)	100 (92.6-100)	100 (78.2-100)	70.6 (58.3-81.0)
	1.79	82.86 (66.4-93.4)	87.5 (74.8-95.3)	82.86 (66.4-93.4)	87.5 (74.8-95.3)
	0.36	88.57 (73.3-96.8)	70.83 (55.9-83.1)	68.89 (53.4-81.8)	89.5 (75.2-97.1)
BAT to sesame	16.11	66.67 (34.9-90.1)	100 (94.9-100)	100 (63.1-100)	94.67 (86.9-98.5)
	8.15	91.67 (61.5-99.8)	98.59 (92.4-100)	91.67 (61.5-99.8)	98.59 (92.4-100)
	14.26	75 (42.8-94.5)	98.59 (92.4-100)	90 (55.6-99.8)	95.89 (88.5-99.1)
BAT to almond	37.57	33.33 (0.8-90.6)	100 (95.5-100)	100 (2.5-100)	97.56 (91.5-99.7)
	0.825	100 (29.2-100)	80 (69.6-88.1)	15.79 (3.4-39.6)	100 (94.4-100)
	18.63	33.33 (0.8-90.6)	93.75 (86.0-97.9)	16.67 (0.4-64.1)	97.4 (90.9-99.7)
BAT to peanut	42.11	45.83 (31.4-60.8)	97.14 (85.1-99.9)	95.65 (78.1-99.9)	56.67 (43.2-69.4)
	4.717	81.25 (67.4-91.1)	85.7 (69.7-95.2)	88.64 (75.4-96.2)	76.92 (60.7-88.9)
	0.124	93.75 (82.8-98.7)	37.14 (21.5-55.1)	67.16 (54.6-78.2)	81.25 (54.4-96.0)
BAT to Ara h 1	16.02	39.58 (25.8-54.7)	97.14 (85.1-99.9)	95.00 (75.1-99.9)	53.97 (40.9-66.6)
	0.82	64.58 (49.5-77.8)	85.71 (69.7-95.2)	86.11 (70.5-95.3)	63.83 (48.5-77.3)
	0.005	79.17 (65.0-89.5)	60.00 (42.1-76.1)	73.08 (59.0-84.4)	67.74 (48.6-83.3)
BAT to Ara h 2	2.264	79.17 (65.0-89.5)	94.29 (80.8-99.3)	95 (83.1-99.4)	76.74 (61.4-88.2)
	0.57	83.33 (69.8-92.5)	91.43 (76.9-98.2)	93.02 (80.9-98.5)	80 (64.4-91.0)
	0.375	85.42 (72.2-93.9)	85.71 (69.7-95.2)	89.13 (76.4-96.4)	81.08 (64.8-92.0)
BAT to Ara h 6	26.71	43.75 (29.5-58.8)	97.14 (85.1-99.9)	95.45 (77.2-99.9)	55.74 (42.5-68.5)
	0.96	72.92 (58.2-84.7)	88.57 (73.3-96.8)	89.74 (75.8-97.1)	70.45 (54.8-83.2)
	0.325	79.17 (65.0-89.5)	74.29 (56.7-87.5)	80.85 (66.7-90.9)	72.22 (54.8-85.8)

*BAT*, Basophil activation test; *CI*, confidence interval.

Optimal concentrations of allergen were 22.73 ng/mL for peanut, 45.45 ng/mL for Ara h 1, 4.55 ng/mL for Ara h 2, 0.91 ng/mL for Ara h 6, 4.545 ng/mL for hazelnut, 22.73 ng/mL for cashew, 113.64 ng/mL for almond, and 113.64 ng/mL for sesame.

**Figure E2:**
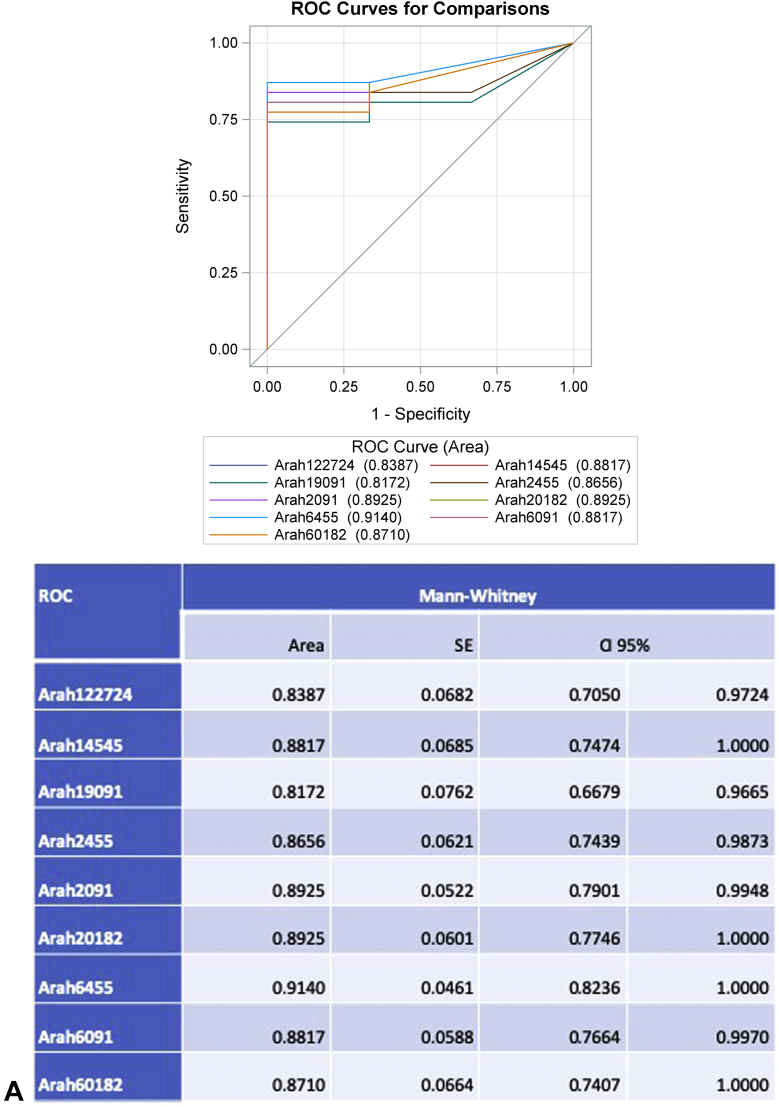

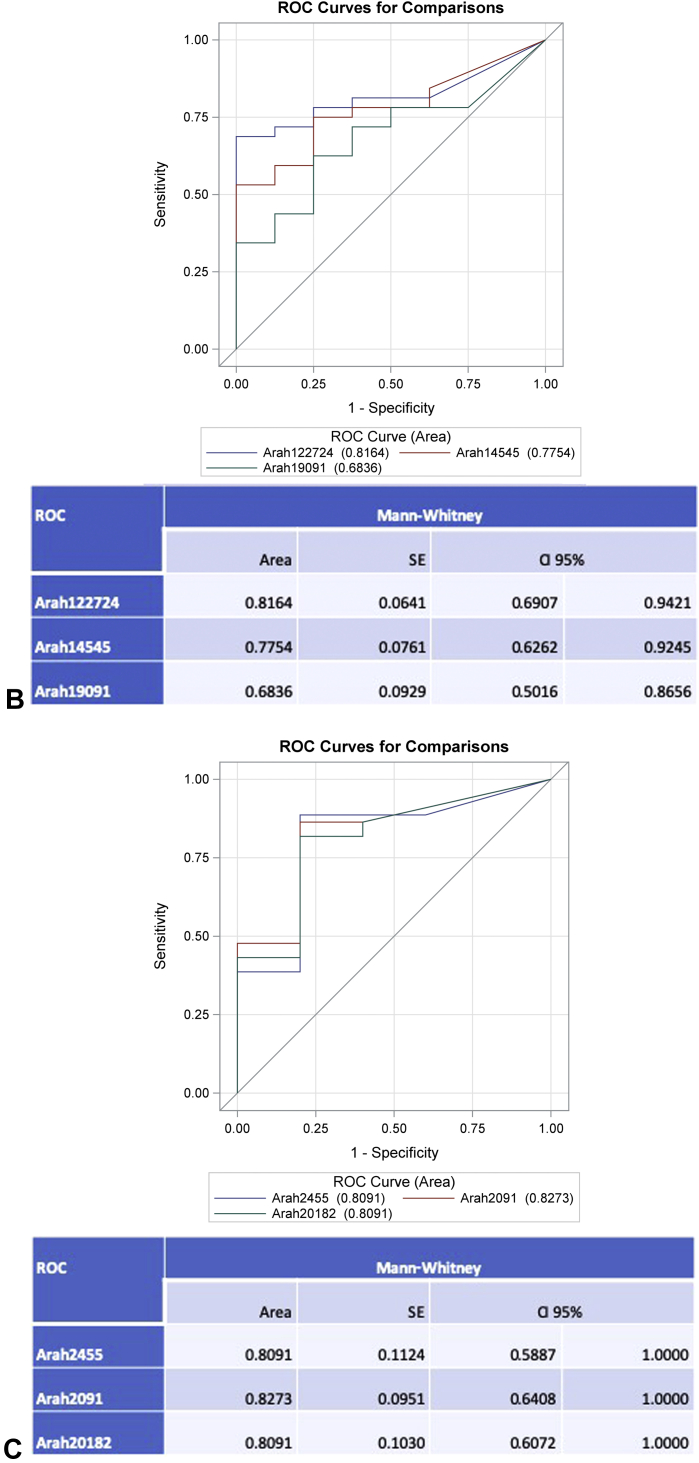
ROC curves for the basophil activation test (BAT) to Ara h 1, Ara h 2, and Ara h 6 for patients who were sensitized to these allergens. (**A**) Patients with specific IgE to Ara h 1 AND Ara h 2 ≥ 0.1 (n = 34). (**B**) Patients with specific IgE to Ara h 1 ≥ 0.1 (n = 40). (**C**) Patients with specific IgE to Ara h 2 ≥ 0.1 (n = 49). *ROC*, Receiver operating characteristic.

For the BAT to peanut components, we also looked at the diagnostic performance in patients who were sensitized to the respective components and these were generally superior to the performance of the same tests in the whole population (Figure E2, available in this article's Online Repository at www.jaci-inpractice.org).

### Basophil activation test as a second step in the diagnostic workup reduces the number of oral food challenges

**Figure E3:**
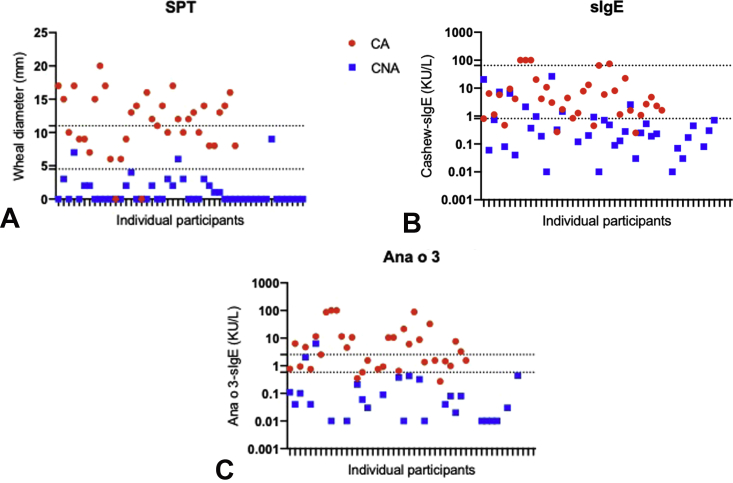
Individual results for cashew SPT (**A**) and specific IgE to cashew (**B**) and to Ana o 3 (**C**) for cashew nut allergic (CA, in red) and cashew nut nonallergic (CNA, in blue). The dotted horizontal lines indicate the negative and positive cutoffs, that is, with 95% positive predictive value and 95% negative predictive value for each test. *sIgE*, Specific IgE; *SPT*, skin prick testing.

Given the high specificity of the BAT, which complements the high sensitivity of SPT and sIgE, and the practicalities involved in the performance of the BAT, which requires fresh blood processed soon after collection and flow cytometry, we had proposed, in a previous study,^11^ that the BAT would be most useful as a second step in the diagnostic workup for peanut allergy, performed in patients with equivocal results for SPT and sIgE to clarify the allergic status. Patients with the positive BAT would have confirmed peanut allergy and patients with a BAT result below the positive cutoff (ie, negative or intermediate BAT) or nonresponder basophils would need an OFC. We assessed the impact of this approach in the number of OFCs not only to peanut but also to the other nuts and seeds assessed on the BAT (Table E3, available in this article's Online Repository at www.jaci-inpractice.org). The cutoffs indicate positive, optimal, and negative cutoffs for SPT, sIgE to whole extract, and components with the respective sensitivity, specificity, and predictive values. Patients with results greater than or equal to the 95% PPV cutoff were considered allergic, patients with results lower than the 95% NPV cutoff were considered nonallergic, and the patients with any combination of the 2 or with results that fell between the 95% PPV and 95% NPV cutoffs were considered equivocal. See Figure E3 in this article's Online Repository at www.jaci-inpractice.org for a graphical representation of the cutoffs and allergic status to cashew nut, as an example.

**Figure 4:**
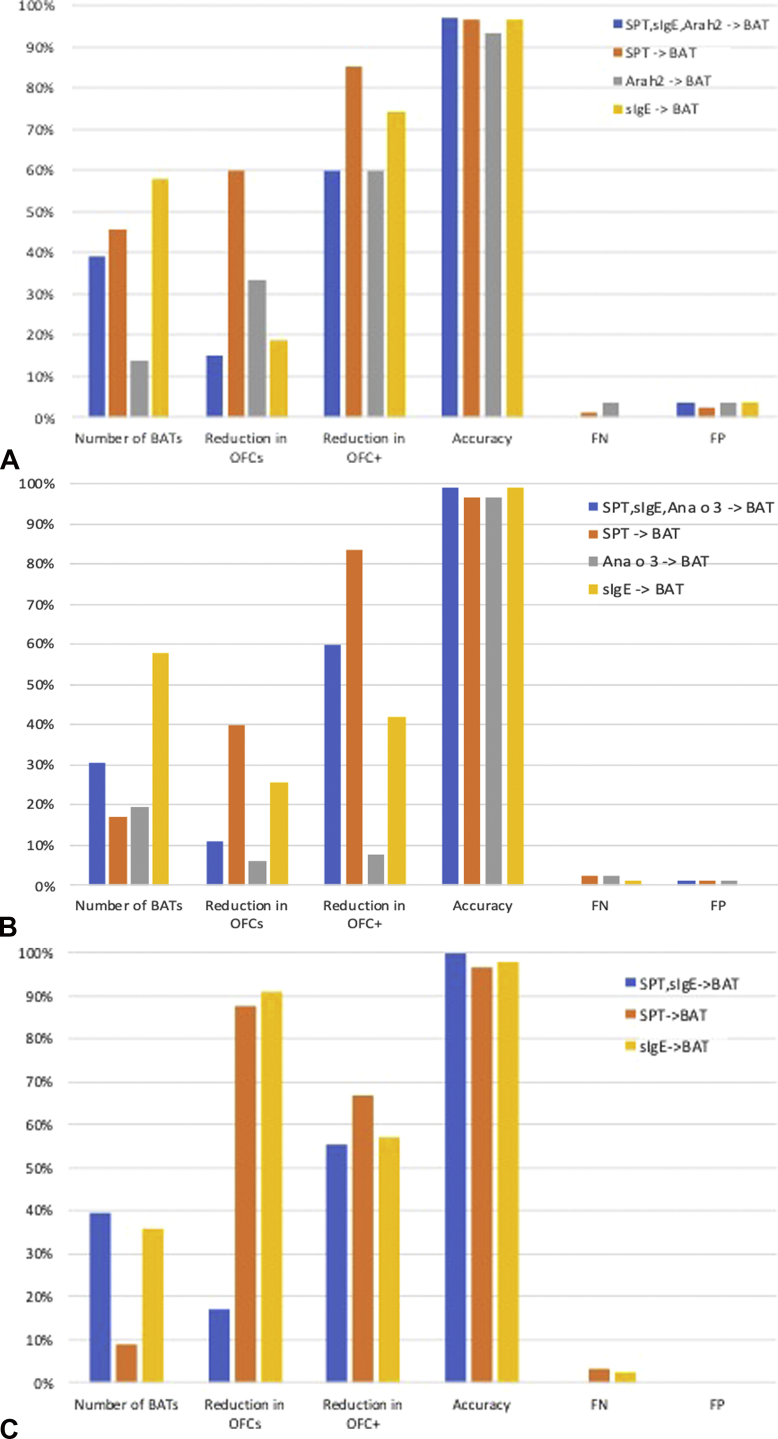
Impact of the basophil activation test (BAT) as a second step in the diagnostic workup after a first step consisting of SPT, specific IgE to the extract, and specific IgE to the best component (Ara h 2 for peanut, Cor a 14 for hazelnut, and Ana o 3 for cashew nut), SPT only, specific IgE only, or specific IgE to the best component only. (**A**) Peanut. (**B**) Cashew nut. (**C**) Sesame seed. *FN*, False-negative; *FP*, false-positive; *OFC*, oral food challenge; *SPT*, skin prick testing.

The diagnostic accuracy and resulting number of OFCs following this approach (ie, a first step consisting of SPT and sIgE and a second step consisting of the BAT) are represented in Table IV for participants with equivocal combination of SPT, sIgE to extracts, and sIgE to individual allergens or components. Figure 4 shows similar figures for SPT followed by the BAT, sIgE to extracts followed by the BAT, and sIgE to components followed by the BAT. Globally, the approach of using the BAT as a second step in the diagnostic workup for nut and seed allergies had a 97% to 100% accuracy with 0% false-negatives and ensured a 5% to 15% reduction in the number of OFCs, except for the BAT to hazelnut. The reduction in positive OFCs seen with this approach ranged between 50% and 75% for the same nuts, thus sparing children from experiencing uncomfortable and potentially severe allergic reactions.

**Table IV:** Testing the proposed approach to using the basophil activation test to diagnose nut and sesame seed allergies

	Outcome of history SPT, sIgE to extracts and components			Misdiagnosis		Outcome of BAT			Outcome of OFC and misdiagnosis		Correct diagnosis—% total patients		Nr BATs required—% total patients		Total OFCs—with BAT (without BAT) and % reduction		Positive OFCs with BAT (without BAT) and % reduction	
Cashew nut allergy	NA	**29**	13	**FN = 0**	FN = 0													
	Equivocal	**27**	17			NR or intermediate	**8**	6	**2** 1	**CA**	**99%**	98%	**31%**	29%	**11%**	6%	**75%**	50%
						Negative	**16**	10	**FN = 0**	0	**87/88**	57/58	**27**	17	**24 (27)**	16 (17)	**2 (8)**	1 (2)
						Positive	**3**	1	**FP = 0**	0								
	Allergic	**32**	28	**FP = 1**	FP = 1													
Sesame seed allergy	NA	**50**	14	**FN = 0**	FN = 0													
	Equivocal	**35**	13			NR or intermediate	**4**	2	**1**	1 **SA**	**99%**	100%	**39%**	45%	**11%**	15%	**50%**	50%
						Negative	**27**	8	**FN = 3**	2	**88/89**	29/29	**35**	13	**31 (35)**	11 (13)	**4 (8)**	3 (6)
						Positive	**4**	2	**FP = 1**	0								
	Allergic	**4**	2	**FP = 0**	FP = 0													
Almond allergy	NA	**69**	40	**FN = 0**	FN = 0													
	Equivocal	**19**	17			NR or intermediate	**4**	4	***All***	***NA***	**100%**	100%	**21%**	29%	**5%**	6%	**75%**	50%
						Negative	**14**	12	**FN = 1**	1	**89/89**	58/58	**19**	17	**18(19)**	16 (17)	**1 (4)**	1 (2)
						Positive	**1**	1	**FP = 0**	0								
	Allergic	**1**	1	**FP = 0**	FP = 0													
Hazelnut allergy	NA	**17**	7	**FN = 0**	FN = 0													
	Equivocal	**59**	38			NR or intermediate	**32**	22	**15**	12 **HA**	**98%**	100%	**67%**	70%	**0%**	0%	**0%**	0%
						Negative	**27**	16	**FN = 1**	1	**86/88**	54/54	**59**	38	**59 (59)**	38 (38)	**15 (15)**	13 (13)
						Positive	**0**	0	**FP = 0**	0								
	Allergic	**12**	9	**FP = 0**	FP = 1													
Peanut allergy	NA	**9**	2	**FN = 0**	FN = 0													
	Equivocal	**34**	17			NR or intermediate	**7**	3	***All***	***NA***	**97%**	96%	**39%**	36%	**15%**	12%	**60%**	33%
						Negative	**22**	12	**FN = 2**	2	**85/88**	45/47	**34**	17	**29 (34)**	15 (17)	**2 (5)**	2 (3)
						Positive	**5**	2	**FP = 2**	1								
	Allergic	45	28	**FP = 1**	FP = 1													

*BAT*, Basophil activation test; *CA*, cashew nut allergic; *FN*, false-negative; *FP*, false-positive; *HA*, hazelnut allergic; *NR*, nonresponder; *OFC*, oral food challenge; *SA*, sesame seed allergic; *sIgE*, specific IgE; *SPT*, skin prick testing.

Numbers in bold indicate the results for the whole population, and numbers in italic refer to the subgroup who were actually challenged to the individual nuts as part of the Pronuts study. Allergic patients had results at or above the 95% positive predictive value (PPV) cutoff or a combination of above the 95% negative predictive value (NPV) and above the 95% PPV; nonallergics had below the 95% NPV for all tests; and equivocal were the remaining cases.

Subjects with results for all tests were included, including subjects with nonresponder basophils: n = 88 for hazelnut, n = 88 for peanut, n = 88 for cashew, n = 89 for almond, n = 89 for sesame.

## Discussion

Avoiding nuts and seeds can have a significant negative impact on the quality of life and mental health of allergic children and their families. The majority of children with nut or seed allergies can tolerate other nuts in their diet, and motivated and informed families can be recommended selective nut eating, while avoiding the culprit nuts to which the child is allergic. The Pronuts study confirmed that the introduction of nuts/seeds in the diet of children with 1 or more nut allergies is feasible and safe^2^; however, this may require multiple OFCs given that IgE sensitization to multiple nuts and seeds is common in nut allergic children. OFCs can be stressful for patients and families and can potentially cause allergic reactions of unpredictable severity. The BAT has shown to have high specificity to diagnose peanut allergy in previous studies and can be used as a second step in the diagnostic workup of food allergy.^11^ We applied this concept to participants in the Pronuts study, who had 1 or more nut/seed allergies, and were being assessed for possible allergy to the other nuts and sesame. We found that the diagnostic performance of the BAT and the other tests varied between nuts/seed, but generally the BAT distinguished well between allergic and nonallergic children, among children with 1 or more allergies to nuts or sesame. Although not statistically significant except for cashew, the area under the ROC curve for the BAT was larger than the ones of the other available tests in the diagnosis of sesame and almond, similar for hazelnut and lower for peanut and cashew nut allergies (Figure 3). The BAT to Ara h 2 was better than the BAT to peanut, Ara h 1, or Ara h 6. The performance of the BAT to peanut components was even better when only children sensitized to that specific allergen, further supporting the use of the BAT as a second-line test when IgE sensitization could not support a definitive diagnosis. When applied as a second step in the diagnostic workup, the BAT had 96% to 100% diagnostic accuracy and allowed a reduction in OFCs, particularly of positive OFCs (except for hazelnut), thus rendering the diagnosis of food allergy at the same time accurate, safe, and more comfortable for children with suspected nut/seed allergies.

Performing the BAT only in patients with an equivocal diagnosis after clinical history, SPT, and sIgE and performing an OFC in patients with negative or equivocal BAT results, for example, between positive and negative cutoffs or nonresponder basophils, allowed a reduction in patients experiencing allergic reactions during the OFC. This reduction varied between 50% and 75% in the whole population of patients tested and between 33% and 50% for the subgroup of patients who underwent an OFC as part of the Pronuts study protocol; except for hazelnut allergy, for which the BAT did not make a difference in the number of OFCs, probably because its diagnostic performance was very similar to that of the other tests. These high percentages of reduction in OFCs relate, however, to small event numbers and therefore may have a lower impact in terms of patient numbers, depending on the scale on which the BAT is applied in clinical practice. Adopting the same approach of performing the BAT as a second step, after only SPT or only sIgE, also enabled a reduction in OFCs, particularly positive OFCs. Generally, performing SPT and BAT was better than performing sIgE and BAT (except for sesame), enabling the greatest reduction in OFCs; however, these approaches with fewer tests resulted in a small proportion of false-positives and false-negatives. The false-negatives are the most concerning as they can result in accidental reactions in the community, which are potentially severe. Performing all tests reduced the false-negatives to zero but often led to more OFCs overall. From a practical point of view, it is important to note that we collected blood for the BAT immediately after SPT in the majority of patients and that the same sequence was followed in previous studies.^11^^,^^12^ Although blood for the BAT should not be collected after *in vivo* procedures with a significant risk of systemic allergic reactions, such as intradermal tests and provocation tests, SPT to foods did not seem to affect BAT performance. Tahini was used for sesame SPT as this contains fat and lipophilic allergens that are often not represented in defatted allergen extracts. A recent study demonstrated that using both extract and tahini paste leads to a better combination of sensitivity and specificity, with the extract providing higher specificity and tahini providing higher sensitivity.^17^

The overlap in BAT results between allergic and nonallergic subjects was smaller for sesame, reflecting the superior diagnostic accuracy of the BAT to sesame compared with the BAT to peanut or tree nuts. The performance of the BAT to peanut in absolute terms was not as good as previously reported by us.^11^ Differences in the BAT methodology between the 2 studies are likely to have accounted for this discrepancy, as the patient population is similar, particularly in the London site, and the performance of the other tests, namely SPT and Ara h 2-sIgE, is comparable in both studies. Different methods for performing the BAT have been described, and the methodology adopted can have an impact on the results, from the laboratory procedure to flow cytometry and data analyses.^14^^,^^18^^,^^19^ Aspects of the methodology to consider are the markers chosen to identify the basophil population, the fluorochromes used, the allergen extract preparations, the allergen concentration selected, and the anticoagulant used for blood collection. EDTA chelates calcium and therefore prevents the calcium influx into the basophils required for degranulation,^20^ which has advantages for stabilization of samples before testing but requires the addition of calcium at the time of the BAT experiment in a given concentration, which may or may not correspond to the physiological concentration of individual patients. These are some of the aspects to consider if a methodological study is to be performed; however, only a head-to-head comparison of both BAT methods would allow us to confirm the real impact of different BAT methodologies. The BAT performance for hazelnut and cashew reported in the Nutcracker study was apparently better^21^; however, differences in the patient population may have contributed to this as in the Nutcracker study only patients who had no history of reaction to the nut were challenged and thus it is possible that more highly allergic (who were not challenged) patients with higher results for the BAT were included, allowing a better discrimination between allergic and nonallergic subjects.

We found that the performance of the BAT to Ara h 2 was superior to that of the BAT to peanut extract, Ara h 1, or Ara h 6. This reflects the superior diagnostic discriminative ability of Ara h 2 compared with the other allergen preparations, particularly compared with peanut extract and Ara h 1, as previously shown for serologic tests.^11^^,^^22^ We have demonstrated the dominance of Ara h 2 also over Ara h 6 in a recently published study using IgE binding and inhibition assays and cellular effector assays.^23^ In our previous BAT to peanut study,^11^ which we have recently validated using the same BAT methodology in a very large population,^24^ we did not perform the BAT to Ara h 2, but it would be challenging to have improved the diagnostic utility of the BAT to peanut in our previous study, which had sensitivity and specificity already above 95%. The disadvantage of using a single allergen in the BAT, as opposed to the whole extract, is that some allergic patients may not be sensitized to that individual allergen, potentially resulting in a false-negative test. On the contrary, the BAT may become more specific, as may have been the case if we had performed the BAT to Cor a 14 alongside hazelnut in the present study, given that the BAT to hazelnut had quite a few false-positives, possibly due to sensitization to PR-10 proteins secondary to tree pollen allergy.

The Pronuts study constitutes a discovery cohort, and our findings need to be validated in an independent cohort. The cutoffs generated are likely to be suited to the population with a similar (high) prevalence of nut allergies, as expected in patients seen in a specialized allergy clinic. Once validated, this approach could be very useful for clinicians evaluating polysensitized children with suspected peanut, tree nut, and sesame seed allergies. Attention should be given to extrapolate these cutoffs only to populations that are similar to the Pronuts study population.

In summary, the BAT can potentially be very helpful in the management of children with 1 or more nut allergies to identify the safe nuts that can be introduced in the diet. As the BAT is very specific in confirming nut and seed allergies, it may reduce the number of patients who experience allergic reactions during OFCs, thus improving the safety profile of this procedure and opening up room for other indications for OFCs, namely educational and psychotherapeutic purposes. In the future, external validation of our findings in independent cohorts and standardization of the methodology are required so that their reliable and consistent clinical application can be broadened and used to improve the care of a larger number of children with suspected food allergies.

## Figures and Tables

**Table E1:** Allergen dilutions performed using reagent and guidance provided by BÜHLMANN

						Conc (ng/mL)	Final Conc (ng/mL)
Peanut BAG-F13	25	ng/vial	μL		Stock	Stimul.	
c1	F13 c1	Reconstitute with	250	CCR-STB	100	22.73	
c2	F13 c2	50	μL of c1 +	200	CCR-STB	20	4.55
c3	F13 c3	50	μL of c2 +	200	CCR-STB	4	0.909
Ara h 1 BAG2-ARAH1	250	ng/vial	μL		Stock	Stimul.	
c1	ARAH1 c1	Reconstitute with	250	CCR-STB	1000	227.27	
c2	ARAH1 c2	50	μL of c1 +	200	CCR-STB	200	45.45
c3	ARAH1 c3	50	μL of c2 +	200	CCR-STB	40	9.091
Ara h 2 BAG2-ARAH2	5	ng/vial	μL		Stock	Stimul.	
c1	ARAH2 c1	Reconstitute with	250	CCR-STB	20	4.55	
c2	ARAH2 c2	50	μL of c1 +	200	CCR-STB	4	0.91
c3	ARAH2 c3	50	μL of c2 +	200	CCR-STB	0.8	0.182
Ara h 6 BAG2-ARAH6	5	ng/vial	μL		Stock	Stimul.	
c1	ARAH6 c1	Reconstitute with	250	CCR-STB	20	4.55	
c2	ARAH6 c2	50	μL of c1 +	200	CCR-STB	4	0.909
c3	ARAH6 c3	50	μL of c2 +	200	CCR-STB	0.8	0.1818
Hazelnut BAG-F17	25	ng/vial	μL		Stock	Stimul.	
c0	F17 c0	Reconstitute with	50	CCR-STB	500	113.64	
c1	F17 c1	Reconstitute with	250	CCR-STB	100	22.73	
c2	F17 c2	50	μL of c1 +	200	CCR-STB	20	4.545
c3	F17 c3	50	μL of c2 +	200	CCR-STB	4	0.9091
Almond BAG-F20	25	ng/vial	μL		Stock	Stimul.	
c0	F20 c0	Reconstitute with	50	CCR-STB	500	113.64	
c1	F20 c1	Reconstitute with	250	CCR-STB	100	22.73	
c2	F20 c2	50	μL of c1 +	200	CCR-STB	20	4.545
c3	F20 c3	50	μL of c2 +	200	CCR-STB	4	0.9091
Cashew BAG-F202	25	ng/vial	μL		Stock	Stimul.	
c0	F202 c0	Reconstitute with	50	CCR-STB	500	113.64	
c1	F202 c1	Reconstitute with	250	CCR-STB	100	22.73	
c2	F202 c2	50	μL of c1 +	200	CCR-STB	20	4.545
c3	F202 c3	50	μL of c2 +	200	CCR-STB	4	0.9091
Sesame BAG-F10	25	ng/vial	μL		Stock	Stimul.	
c0	F10 c0	Reconstitute with	50	CCR-STB	500	113.64	
c1	F10 c1	Reconstitute with	250	CCR-STB	100	22.73	
c2	F10 c2	50	μL of c1 +	200	CCR-STB	20	4.545
c3	F10 c3	50	μL of c2 +	200	CCR-STB	4	0.9091

Conc, Concentration.

c0-c3 represent the sequential dilutions of stimulant. “BAG2…” indicates the name of the allergen as commercialized by BÜHLMANN. “CCR-STB” is the stimulation buffer included in the Flow CAST kit.

**Table E2:** Demographic and clinical characteristics of participants with nonresponder basophils and participants with responder basophils included in the ROC curve analyses

	Summary statistics	Total (n = 90)	Population included (n = 83)	Nonresponders (n = 7)	*P* value (N = 83 vs 7)
Age (y)	Median (IQR)	5.1 (3-9)	5.2 (3-9)	2.8 (1-13)	.378
Gender (male)	%, n/Pts	54.4 (49/90)	56.6 (47/83)	28.6 (2/7)	.239
Atopic dermatitis (AD)	%, n/Pts	61.1 (55/90)	60.2 (50/83)	71.4 (5/7)	.701
History of AD	%, n/Pts	83.9 (73/87)	83.8 (67/80)	85.7 (6/7)	1.000
Allergic rhinoconjunctivitis	%, n/Pts	46.7 (42/90)	47.0 (39/83)	42.9 (3/7)	1.000
Asthma	%, n/Pts	32.2 (29/90)	34.9 (29/83)	0.0 (0/7)	.092
Allergic to other food	%, n/Pts	41.1 (37/90)	39.8 (33/83)	57.1 (4/7)	.440

*IQR*, Interquartile range; *ROC*, receiver operating characteristic.

**Table E3:** Positive, negative, and optimal cutoffs for the various tests in this population used to determine the need for oral food challenges represented in Table IV

Allergen	Test	Cutoff	Sensitivity	Specificity	Positive predictive value	Negative predictive value	Accuracy
Hazelnut	SPT	19.00	23.08	100.00	100.00	74.03	79.02
		3.00	92.31	73.68	61.54	95.45	78.76
		2.50	96.15	70.18	59.52	97.56	77.26
	sIgE	51.80	16.00	98.25	80.00	72.73	75.82
		0.43	96.00	45.61	43.64	96.30	59.35
		1.34	88.00	63.16	51.16	92.31	69.93
	Cor a 14	6.22	37.50	100.00	100.00	79.17	82.95
		0.01	95.83	40.35	40.35	95.83	55.48
		1.15	70.83	92.98	80.95	88.33	86.94
Peanut	SPT	16	43.75	97.14	95.45	55.74	68.62
		2	97.92	62.86	78.33	95.65	81.59
		5	97.92	82.86	88.68	96.67	90.90
	sIgE	16.4	46.81	97.06	95.65	56.9	70.22
		0.09	100	32.35	67.14	100	68.48
		4.271	74.47	91.18	92.11	72.09	82.26
	Ara h 2	0.29	87.5	94.12	95.45	84.21	90.58
		0.04	95.83	73.53	83.64	92.59	85.44
		0.64	85.42	100	100	82.93	92.21
Cashew	SPT	11	74.29	97.92	96.3	83.93	88.25
		2.5	94.29	81.25	78.57	95.12	86.58
		4.501	91.43	93.75	91.43	93.75	92.80
	sIgE	64.69	14.29	100	100	61.04	64.94
		0.27	97.14	59.57	64.15	96.55	74.94
		0.81	88.57	80.85	77.5	90.48	84.01
	Ana o 3	2.53	55.88	97.87	95	75.41	80.69
		0.38	94.12	89.36	86.49	95.45	91.31
		0.58	94.12	95.74	94.12	95.74	95.08
Almond	SPT	12	66.67	97.5	50	98.73	96.81
		12	66.67	97.5	50	98.73	96.81
		1.5	100	66.25	10	100	67.01
	sIgE	1.544	100	79.75	15.79	100	80.21
		1.544	100	79.75	15.79	100	80.21
		1.544	100	79.75	15.79	100	80.21
Sesame	SPT	16	50	100	100	92.21	95.51
		6	75	94.37	69.23	95.71	92.63
		4	83.33	94.37	71.43	97.1	93.38
	sIgE	50.87	25	100	100	88.61	93.26
		0.92	83.33	67.14	30.3	95.92	68.60
		0.85	91.67	67.14	32.35	97.92	69.34

*sIgE*, Specific IgE; *SPT*, skin prick testing.
